# S178. ALTERED GYRIFICATION IN THE SCHIZOPHRENIA SPECTRUM

**DOI:** 10.1093/schbul/sby018.965

**Published:** 2018-04-01

**Authors:** Daiki Sasabayashi, Yoichiro Takayanagi, Tsutomu Takahashi, Yusuke Takegoshi, Tokio Matsuda, Atsushi Furuichi, Mikio Kido, Yumiko Nishikawa, Mihoko Nakamura, Kyo Noguchi, Michio Suzuki

**Affiliations:** 1 University of Toyama Graduate School of Medicine and Pharmaceutical Sciences; 2 University of Toyama

## Abstract

**Background:**

Increased gyrification in diverse cortical areas has been reported in patients with schizophrenia, which is considered to reflect deviations in early neurodevelopment. Schizotypal personality disorder (SPD) is thought to be a prototypic disorder within the schizophrenia spectrum, which shares biological and psychological commonalities with schizophrenia as a neurobiological basis for vulnerability factors. However, to the best of our knowledge, no magnetic resonance imaging (MRI) studies have investigated the gyrification pattern in SPD.

**Methods:**

T1-weighted structural MRI scans were obtained by 1.5-T scanner from 101 patients with schizophrenia, 46 patients with SPD, and 77 age- and gender- matched healthy control subjects. Using FreeSurfer software (version 5.3.), the local gyrification indices (LGIs) of entire cortex were obtained with the method of Schaer and colleagues. Clinical symptoms of the patients were rated with the Scale for the Assessment of Positive Symptoms (SAPS) and the Scale for the Assessment of Negative Symptoms (SANS) at the time of scanning. A general linear model controlling for age, gender, medication dose, and duration of medication was used to compare the LGIs across the groups and to conduct vertex-by-vertex whole brain LGI correlation analyses with clinical variables. This study was approved by the Committee on the Medical Ethics of Toyama University based on the declaration of Helsinki. After a complete description of the study was provided, written informed consent was obtained from all subjects.

**Results:**

Compared with the controls, the patients with schizophrenia showed significantly higher LGI in widespread cortical areas including the bilateral frontal, parietal, and occipital regions. The patients with SPD demonstrated significantly higher LGI in the bilateral frontal and left parietal regions compared with the controls. Compared with the patients with SPD, the patients with schizophrenia showed significantly higher LGI in the left occipital and right frontal regions. Both SAPS and SANS total scores were positively correlated with LGI in the bilateral temporal regions in patients with schizophrenia, and were negatively correlated with LGI in the bilateral occipital regions in patients with SPD.

**Discussion:**

Increased LGI in the bilateral frontal regions may be the common morphological substrates for the schizophrenia spectrum, possibly representing vulnerability to schizophrenia. In addition, increased LGI in the left occipital and right frontal regions preferentially observed in schizophrenia may have a critical role in manifestation of florid psychotic symptoms.

